# The complete chloroplast genome of *Toona sinensis*, an important economic and medicinal plant endemic in China

**DOI:** 10.1080/23802359.2021.1895691

**Published:** 2021-03-18

**Authors:** Li Xiang, Lushui Zhang, Jinyao Hu

**Affiliations:** a Economics Institute of Southwest Minzu University, Chengdu, China; b Research Center for Forest and Grassland Disaster Prevention and Reduction, Mianyang Normal University, Mianyang, China

**Keywords:** *Toona sinensis*, chloroplast genome, endemic species, phylogenetic

## Abstract

*Toona sinensis* is an economic and medicinal plant endemic in China. In this study, the complete chloroplast genome of *T. sinensis* was assembled using the second-generation high-throughput sequencing data. The genome consists of 138 genes in total, including 89 protein-coding genes, 7 ribosomal RNA genes, 40 transfer RNA genes and 2 pseudogenes. Phylogenetic analysis indicated that *T. sinensis* has a close relationship with the *Toona ciliata* with strong support. The chloroplast genome presented here provides a valuable resource to conserve this valuable species.

*Toona sinensis* (A.Juss.) M.Roem., belonging to *Toona* Roem in Meliaceae family, is an important economic and medicinal species. The whole body of *T. sinensis* is full of treasure. The young and tender leaves are aromatic palatable and can make many kinds of characteristic recipes, the main stem is an excellent wood for furniture, interior decoration and shipbuilding, the roots, barks, and fruits can be used as medicine to stringent, stop bleeding, remove dampness, and relieve pain (Xing and Chen 2010; Chen et al. 2013; Hu et al. 2016; Peng et al. 2019; Wang et al. 2020). In China, genus *Toona*, especially *T. sinensis* has a long cultivation history, because of its great development value and utilization potential, it is often used as a resource plant and widely promoted (Peng and Liang 2003). Previous studies have shown that *T. sinensis* belongs to the genus *Toona* with unquestioned. However, there is still controversy on the classification of *Toona* varieties according to the allelic enzyme analysis (Lu et al. 2001). Liu et al. (2019) reported the complete chloroplast genome of *T. sinensis.* Much remained unknown about the complete chloroplast genome of *T. sinensis.* In this study, we assembled the complete chloroplast genome of *T. sinensis* once more used its wild individual and performed a phylogenetic analysis with 20 other species based on their complete chloroplast genomes, which might improve an appreciation of its genetics that would be conducive to the formulation of conservation and management strategies of this species.

We collected fresh leaves of a wild *T. sinensis* individual from Beichuan Qiang Autonomous County in Sichuan Province, China (31.481513 N, 104.265168E). Voucher specimen (MNU-PHO-1226) of the species was stored in the Ecological Security and Protection Key Laboratory of Sichuan Province, China. The total DNA was extracted with the DNA-secure Plant Kit (TIANGEN). We performed the whole-genome sequencing with the BGISEQ-500 Platform (BGI, China) and obtained about 10 Gb high-quality clean data for the subsequent analysis. The complete chloroplast genome of *T. sinensis* was *de novo* assembled with NOVOPlasty v.4.1 (Dierckxsens et al. 2017). We used Geneious v.8.1.4 (Kearse et al. 2012) to compare and adjust the assembled complete chloroplast sequence manually. The gene prediction was carried out by Plann (Huang and Cronk 2015) and checked the quality with Sequin v.15.50 (Clark et al. 2016). Finally, we obtained a chloroplast genome of *T. sinensis.* The genome has been both submitted to the Genome Warehouse in National Genomics Data Center (Zhang et al. 2020) and the GenBank, under the accession number of GWHAZIK00000000 and MW401816, respectively.

The complete chloroplast of *T. sinensis* is 159,265 bp in length, with a GC content of 37.88% in total. The chloroplast genome of *T. sinensis* contains a total of 138 genes, including 89 protein-coding genes (PCGs), 40 transfer RNA (tRNA) genes, and 7 ribosomal RNA (rRNA) genes and 2 pseudogenes. Most of these genes are single copy genes, while there are 15 genes (9 PCGs, 9 tRNA genes, 3 rRNA genes) were duplicated in the IR regions. Compared with Liu et al. (2019) research results, we assembled a longer genome sequence (159,265 bp vs 157, 228 bp) and annotated more genes (138 vs 126). What is exciting is that we annotated 2 pseudogenes (ycf1 and ycf15). As a new molecular marker technology, ycf15 and ycf1 gene not only has a certain significance of species identification, but also has great potential in the phylogenetic studies (Gao et al. 2017). It will contribute to finishing the controversy on the classification of *T. sinensis* varieties.

To infer the phylogenetic position of *T. sinensis*, we reconstructed a phylogenetic tree using the complete chloroplast genome sequences of *T. sinensis* and other 20 species downloaded from the NCBI (Figure 1). The sequences were aligned using the software MAFFT (Katoh and Standley 2013) and the Maximum Likelihood analysis worked on software RAxML v.8.2.9 (Stamatakis 2014) setting GTRGAMMA as the best model and 1000 bootstrap tests. The phylogenetic tree demonstrates that *T. sinensis* is closely related to *Toona ciliata* M.Roem. with strong support (Figure 1), which is consistent with Liu et al. (2019).

**Figure 1.**
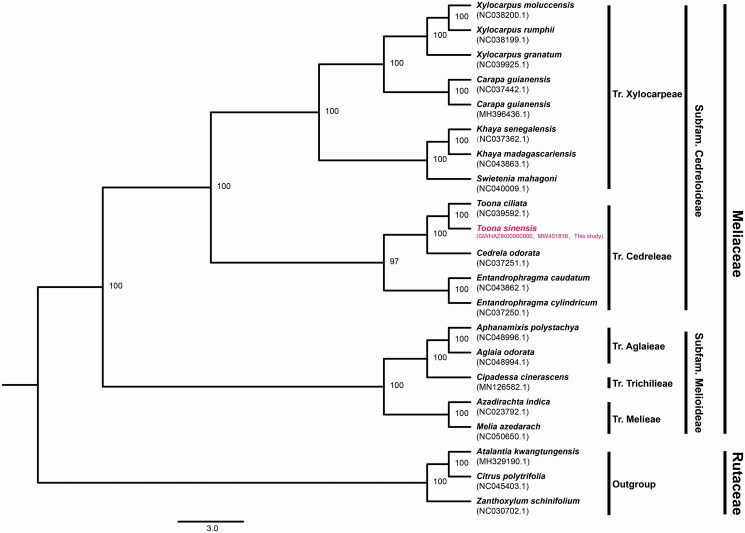
Maximum likelihood phylogenetic tree based on the cp-genome sequences of *Toona sinensis* and other 20 species. Numbers in the nodes are the bootstrap values from 1000 replicates. The numbers in brackets are the NCBI and Genome Warehouse in National Genomics Data Center accession number of each species.

In summary, we provide a valuable genomic information of *T. sinensis* different from the previous, which could help us facilitate the identification, conservation, and utilization of this valuable species. In addition, it is also help for us to understand the phylogenetic relationship of genus *Toona* and even the family Meliaceae.

## Data Availability

The whole genome sequence data reported in this paper has been deposited in the Genome Warehouse in National Genomics Data Center (Zhang et al. 2020), Beijing Institute of Genomics (China National Center for Bioinformation), Chinese Academy of Sciences, under accession number GWHAZIK00000000 that is publicly accessible at https://bigd.big.ac.cn/search/?dbId=gwh&q=GWHAZIK00000000. The associated BioProject, SRA, and Bio-Sample accession numbers are PRJCA004103 (https://bigd.big.ac.cn/bioproject/browse/PRJCA004103), CRA003691 (https://bigd.big.ac.cn/gsa/browse/CRA003691) and SAMC297323 (https://bigd.big.ac.cn/biosample/browse/SAMC297323), respectively. The voucher specimen of the species is free accessible at Ecological Security and Protection Key Laboratory of Sichuan Province (http://zdsys.mnu.cn/, Lushui Zhang, fly155640@163.com), China, under the voucher number MNU-PHO-1226.
